# Genetic factors responsible for eating and cooking qualities of rice grains in a recombinant inbred population of an inter-subspecific cross

**DOI:** 10.1007/s11032-014-0065-8

**Published:** 2014-04-04

**Authors:** Yu-Chia Hsu, Meng-Chun Tseng, Yong-Pei Wu, Meng-Ying Lin, Fu-Jin Wei, Kae-Kang Hwu, Yue-Ie Hsing, Yann-Rong Lin

**Affiliations:** 1 Department of Agronomy, Chiayi Agricultural Experiment Station, Taiwan Agricultural Research Institute, Chiayi, Taiwan; 2 Department of Agronomy, National Taiwan University, No. 1, Sec. 4, Roosevelt Road, Taipei, 10617 Taiwan; 3 Institute of Plant and Microbial Biology, Academia Sinica, Taipei, Taiwan

**Keywords:** Grain quality, Palatability, RVA parameters, Starch synthesis-related gene, Viscosity

## Abstract

The eating and cooking qualities of rice grains are the major determinants of consumer preference and, consequently, the economic value of a specific rice variety. These two qualities are largely determined by the physicochemical properties of the starch, i.e. the starch composition, of the rice grain. In our study, we determined the genetic factors responsible for the physicochemical properties of starch in recombinant inbred lines (RILs) of *japonica* cv. Tainung 78 × *indica* cv. Taichung Sen 17 (TCS 17) cultivated over two crop seasons by examining palatability characteristics and several Rapid Viscosity Analyzer (RVA) parameters. Thirty-four quantitative trait loci (QTLs), each explaining between 1.2 and 78.1 % phenotypic variation, were mapped in clusters on eight chromosomes in 190 RILs genotyped with 139 markers. Ten pairs of QTLs were detected in the two environments, of which seven were in agreement with previous findings, suggesting that these QTLs may express stable experimental populations across various environments. Waxy (*Wx*), which controls amylose synthesis, was determined to be a primary gene regulating the physicochemical properties of cooked rice grains, as indicated by the presence of a major QTL cluster on chromosome 6 and by marker regression analysis. Six starch synthesis-related genes (SSRGs) which were located in the QTL intervals significantly differed in terms of gene expression between the two parents during grain-filling and were important genetic factors affecting physicochemical properties. The expression of four genes, *PUL*, *ISA2*, *GBSSI*, and *SSII*-*3*, was significantly upregulated in TCS 17, and this expression was positively correlated with six traits. The effects of the six SSRGs and gene interaction depended on genetic background and environment; grain quality may be fine tuned by selecting for *SBE4* for *japonica* and *PUL* for *indica*. We provide valuable information for application in the breeding of new rice varieties as daily staple food and for use in industrial manufacturing by marker-assisted selection.

**Electronic supplementary material:**

The online version of this article (doi:10.1007/s11032-014-0065-8) contains supplementary material, which is available to authorized users.

## Introduction

Rice is one of the important cereal crops grown worldwide and is the major stable food for more than one-half of the world’s population, providing approximately 23 % of the daily caloric intake (Fischer et al. 2000). Increasing grain yield has been an important breeding goal because the demand for rice is expected to increase by up to 40 % by 2030 (Khush 2005). However, improving rice grain quality is also imperative because of consumers’ preference, farmers’ profits, and multiple uses in food processing both at home and by industry. Specifically, in Taiwan, where rice is cultivated and harvested in two crop seasons every year, the breeding goal has focused on premium grain quality for the past 40 years. Additionally, rice starch, which accounts for approximately 90 % of the grain contents, is a valuable commodity and has been used in food and pharmaceuticals because of its bland taste, creamy texture, spreadability, and non-allergenicity (Wani et al. 2012). Breeding new varieties with various grain qualities to meet these diverse purposes is therefore an important target worldwide.

There are various aspects to rice grain quality, including grain appearance, milling quality, nutritional quality, and eating and cooking qualities (ECQ); these qualities are very important to consumers and the marketplace (Juliano 1998). Eating quality refers to the sensory perception of consumers of the cooked rice and is associated with such characteristics as glossiness, flavor, and stickiness. These latter characteristics reflect the chemical reaction that occurs during cooking of the rice grain, including hydration, gelatinization, length of cooking time, kernel elongation, and volume expansion (Bhattacharya and Sowbhagya 1971; Juliano and Perez 1984). Evaluating ECQ by assessing these characters is laborious, and a large number of rice samples cannot be screened in a timely manner. Nevertheless, the physicochemical properties of rice starch in endosperm have been used as an indirect index of ECQ. Because the rice grain is mainly composed of starch, apparent amylose content (AAC), gel consistency (GC), and gelatinization temperature (GT) are the three major characters used to assess ECQ (Cagampang et al. 1973; McKenzie and Rutger 1983). Starch viscosity and thermodynamic properties are additional properties that are also used to evaluate grain quality.

Several tools have been developed to assess the physicochemical properties of the rice grain, including palatability (PLS) measured by the Toyo Test Meter and viscosity profiles measure by the Brabender viscograph procedure and Rapid Visco Analyzer (RVA) (Bao and Xia 1999; Deffenbaugh and Walker 1989). Based on hydration retention on the cooked rice grains detected at a given electromagnetic wavelength, the palatability score measured by the Toyo Test Meter is positively correlated with eating quality and used to evaluate rice grain quality (Lestari et al. 2009; Sun et al. 2011). The RVA, a standard tool used in rice breeding programs and industrial manufacturing, simulates the cooking process of rice flour by using “heat–hold–cool–hold” temperature cycles to reveal the pasting properties of rice starch in the cooked grain (Bergman et al. 2004). As palatability—but not pasting properties—is negatively correlated with protein content, ECQ are not directly related to each other and may, therefore, be controlled and modulated by different factors (Lestari et al. 2009; Sun et al. 2011). However, ECQ are complicated, and an easy and standard index to evaluate grain quality is as yet unavailable due to the many diverse aspects that must be considered, although regression models with different physicochemical properties and viscosity have been established to predict the ECQ of cooked grains for *indica* and *japonica* rice, respectively (Wu et al. 2007b).

The physicochemical properties of cooked rice grains revealed by RVA profiles, which delineate a relationship among temperature, viscosity, and time lapse during flour suspension at a given shearing force, are commonly recognized as several characteristics, such as pasting temperature (PaT), peak viscosity (PKV), peak time (PeT), hot paste viscosity (HPV), breakdown viscosity (BDV), cool paste viscosity (CPV), setback viscosity (SBV), and total setback or derived characteristics, such as consistency viscosity (CSV), relative breakdown, setback ratio, and consistency ratio (Bergman et al. 2004). Genetic studies of RVA characteristics in reciprocal crosses of six long-grain rice varieties revealed that PKV, HPV, and CPV were inherited with additive effects and that HPV, CPV, and CSV were inherited with nuclear gene and maternal effects (Bao and Xia 1999). In two subsequent studies, 20 and 26 quantitative trait loci (QTLs) conferring RVA characteristics were identified in the double haploid (DH) population of *indica*/*japonica* and recombinant inbred lines (RILs) of *indica*/*indica*, respectively (Bao et al. 2002; Wang et al. 2007).

A major QTL cluster contributing largely to the phenotypic variation of several viscosity characteristics has been mapped to chromosome 6 corresponding to the* Waxy* (*Wx*) locus. *Wx* encodes* granule-bound starch synthase-I* (*GBSSI*), a key gene determining the ratio of amylose to amylopectin which is the critical factor affecting the ECQ of the cooked rice grain. Six alleles of *Wx* have been found in natural germplasm: *wx* with null function, resulting in glutinous rice with low AC, and *Wx* ^*a*^ and *Wx* ^*b*^, which undergo diversified selection in the two lineages of *indica* and *japonica*, with 20–30 and 15–22 % AC, respectively (Juliano 1992; Liu et al. 2009; Mikami et al. 2008; Yamanaka et al. 2004). A second QTL cluster that predominantly confers alkali spreading has been mapped to chromosome 6; this QTL cluster corresponds to the *Alk* locus. *Alk* was isolated to study its function as the *starch synthase IIa* (*SSIIa*) and found to mainly determine the GT of cooked rice grains (Gao et al. 2003; Umemoto et al. 2002; Zhang et al. 2011). However, pullulanase, encoded by *PUL* and mapped to chromosome 4, can debranch pullulan and amylopectin; *PUL* plays a role in determining the fine structure of amylopectin and influences the variation in RVA profiles of several glutinous rice varieties (Nakamura et al. 1996; Yan et al. 2011).

The quantities and properties of both protein and, in particular, starch are determinants of rice yield and grain quality (Duan and Sun 2005). Many enzymes, some with various isoforms, are involved in starch biosynthesis, with the result being a specific starch composition in the endosperm as the end-product of a complicated process that influences grain quality (Pandey et al. 2012). Among the starch-synthesizing enzymes, *GBSSI* (*Waxy*) and *SSIIa* (*Alk*) are considered to determine rice ECQ by affecting AC, GC, and GT (Tian et al. 2009). Also, starch synthesis-related genes (SSRGs), such as debranching enzyme genes (*DBE*), isoamylase genes (*ISA*), starch branching enzyme genes (*SBE*), soluble starch synthase genes (*SSS*), ADP-glucose pyrophosphorylase large subunit genes (*AGPlar*), *PUL*, and AGP large subunit isoform genes (*AGPiso*) had minor effects on AC, GC, and palatability. The coordinated functioning of these enzymes has been proposed to control AC and GT, which is in accordance with the results of QTL analyses of the physicochemical properties of rice grains (Pandey et al. 2012; Sun et al. 2011; Tian et al. 2009).

ECQ vary according to germplasm of different genetic backgrounds, environment, and gene–environment interactions (Liu et al. 2011; Yan et al. 2011). Many environmental factors, such as climate conditions and agronomic practice, influence gene expression and thereby affect grain formation during grain-filling. For example, high temperature impedes rice grain-filling by disturbing the expression of starch-synthetic genes and SSRGs that are involved in the deposition of storage materials, such as starch, thus resulting in poor grain quality with a chalky appearance and reduced head rice yield (Lur et al. 2009; Yamakawa et al. 2007; Zhao and Fitzgerald 2013). RILs or DH populations cultivated in different environments have been used to identify different QTL clusters for GC, AC, and RVA parameters (Fan et al. 2005; Tian et al. 2005; Wang et al. 2007). Using a chromosome segment substitution line (CSSL) population across eight environments, Liu et al. (2011) were able to commonly map only 42 % (56/132) of QTLs conferring 16 quality traits in at least three environments. However, the same QTLs, such as *Wx* and *Alk*, have been consistently detected from different segregating populations of different cross-combinations across environments (Aluko et al. 2004; Gao et al. 2003; Tian et al. 2005; Umemoto et al. 2002; Wang et al. 2007).

There is a continually increasing demand for improved rice grain quality and grain quality, which vary by germplasm and environment, as determined in terms of the physicochemical properties of rice starch. Therefore, we used a RIL population derived from an inter-subspecific cross cultivated in two environments to identify the expressed QTLs conferring ECQ. Specifically, 190 RILs derived from a cross of two elite cultivars in Taiwan, *japonica* cv. Tainung 78 (TNG 78) and *indica* cv. Taichung Sen 17 (TCS 17), to identify QTLs during two cropping seasons. Our aim was to (1) detect QTLs affecting physicochemical properties segregating in this population; (2) determine different sets of QTLs in different cropping seasons and in given genetic backgrounds (*indica* or *japonica* haplotype); (3) identify genes corresponding to QTLs for physicochemical properties by using the candidate gene approach with verification of differential expression between the two parents during grain-filling. This study provides information that will be useful to elucidate the relationships of ECQ and starch-synthetic genes and/or SSRG and to breed cultivars with various grain qualities for multi-purpose application of *indica* and *japonica* rice by marker-assisted selection.

## Materials and methods

### Development of the mapping population

We established a segregation population by crossing a *japonica* cultivar, TNG 78, and an *indica* cultivar, TCS 17. Cultivars TNG 78 and TCS 17 differ significantly in grain appearance, AC, palatability, gel consistency, crude protein content, and viscosity, and these differences provide the means to clarify the genetic basis of the ECQ of the rice grain. An F_7_ population of 190 RILs was grown in the second crop season of 2010 (2010-II) and the first crop season of 2011 (2011-I) in paddy fields located at Chiayi Agricultural Experiment Station, Chiayi, Taiwan. We randomly selected 24 individuals from each RIL to evaluate the palatability and viscosity of cooked rice grains from each RIL.

### Assessment of palatability and RVA parameters

Rice grains were dehulled and ground into fine flour, and the palatability of samples (approx. 32 g) of rice flour from each RIL was assessed using the palatability analyzer system (Toyo Taste Meter, Model MA-30; TRCM Co., Toyo Rice Polishing Machine Factory, Osaka, Japan). The viscosity of the cooked rice grain was analyzed using the Rapid Visco Analyzer to obtain RVA profiles (Model No. RVA-4; Newport Scientific, Sydney, Australia), according to Standard Method AACC61-02 released by the American Association of Cereal Chemists. Briefly, approximately 3 g of rice flour was mixed with 25 mL water. The sequential temperature curve for a 12.5-min test was as follows: (1) incubation at 50 °C for 1 min; (2) increase in temperature to 95 °C and holding for 2.5 min; (3) cooling to 50 °C and holding at 50 °C until the end of the cycle. RVA profiles were characterized by five parameters: PKV, HPV, CPV, BDV (= PKV − HPV), and SBV (= CPV − PKV). We also recorded PeT and PaT.

### Genotype assay of molecular markers

The rice genomic DNA extraction procedure of Watanabe et al. (1998) was adopted with modification of the minipreparation. Fresh leaf tissue (each sample approx. 0.05 g) from 6- to 8-week-old young seedlings was homogenized with 300 μL extraction buffer (100 mM Tris-HCl, pH 9.0; 40 mM EDTA-2Na, pH 8.0; 1.67 % sodium dodecyl sulfate) at 30 oscillations per second for 2 min in the TissueLyser (Qiagen Retsch GmbH, Hilden, Germany), followed by the addition of 150 μL benzyl chloride and vortexing. After incubation in a 50 °C water bath for 15 min, 150 μL of 3 M sodium acetate (pH 5.2) was added. The supernatants were collected after centrifugation at 15,000 rpm for 15 min, 4 °C, and 300 μL ice-cold isopropanol was then added to each sample of precipitated DNA. After centrifugation at 15,000 rpm for 10 min, DNA pellets were extracted and washed with 70 % ethanol, air-dried, and dissolved in 50 μL TE buffer.

Two types of PCR-based markers, i.e., simple sequence repeats (SSRs) and sequence tagged sites (STSs), were mostly used in the genotype assays; primer sequences have been deposited in the Gramene (http://www.gramene.org/markers/) and Rice Genome Research Program databases (RGP; http://rgp.dna.affrc.go.jp/E/publicdata/caps/index.html). To determine whether the genes involved in starch metabolism affected the physicochemical properties of cooked rice grains, we analyzed nine markers for starch synthesis genes (Yan et al. 2011), of which five, *ISA1* (isoamylase genes 1), *PUL* (pullulanase), *SBE4* (starch branching enzyme genes 4), and *SSII-1* (starch synthesisII-1), displayed polymorphism between TNG 78 and TCS 17. As mentioned, *Wx* modulates AC in *japonica* and *indica* rice. We used the derived cleaved amplified polymorphic sequences (dCAPs) marker based on the functional nucleotide polymorphism (FNP) distinguishing *Wx* ^*a*^ and *Wx* ^*b*^ (Yamanaka et al. 2004) to genotype the parents and RILs. A 10-μL PCR reaction containing 20 ng genomic DNA, 0.2 μM forward and of reverse primers, and 5 μL T*aq* DNA Polymerase Master Mix RED (Ampliqon, Odense M, Denmark) was amplified in a thermocycler (GeneAmp PCR System 9700, Life Technologies Corp., Carlsbad, CA); the cycling program consisted of one cycle of 94 °C for 5 min; 35 cycles of 94 °C for 30 s, 55 °C for 30 s, and 72 °C for 30 s; one cycle of 72 °C for 5 min. In all, 2 μL amplified DNA products was separated on 2.5 % SFR agarose (AMERSCO, Framington, MA) by using Liberty 120 (Biokeystone, Liberty 120) in 1× TAE at 250 V for 9–20 min, depending on the size difference between the amplified DNA fragments.

### Analyses of QTLs conferring palatability and RVA parameters

The linkage map was constructed with 190 RILs genotyped with 133 polymorphic markers of TNG 78/TCS 17 using the MSTmap, with the Kosambi function and crossing-over frequency used to estimate genetic distance between two markers (Wu et al. 2007a). The positions, gene action, and effect of QTLs conferring palatability and seven RVA parameters were analyzed by composite interval mapping (CIM) using Windows QTL Cartographer 2.5 (Wang et al. 2012). Model 6 with a window size of 10 cM was used to scan 12 linkage groups (LGs) by the backward regression method with up to five markers as a background control. A limit of detection (LOD) threshold for declaring the significance of a putative QTL was determined on the basis of 1,000 random permutations of the trait values at *p* ≤ 0.05 (Churchill and Doerge 1994). Six molecular markers corresponding to *ISA1*, *PUL*, *SBE1*, *SBE4*, *SSII*-*1,* and *Wx* [Electronic Supplementary Material (ESM) Table S1] were used to determine the relevance of these genes to physicochemical properties using the marker regression method of the R/qtl package (Broman et al. 2003). A backward elimination scheme was chosen to eliminate minimal nonsignificant components of the model in a stepwise manner, starting from the full model which consisted of all six functional markers and two-way interactions. Allele effects were then estimated based on backward-eliminated models and expressed as the effect of the allele from cv. TNG 78.

### Quantitative real-time PCR analysis of the expression of candidate genes responsible for palatability and viscosity

Next-generation sequencing in a Genome Analyzer II DNA sequencer (Illumina, San Diego, CA) was carried out on cv. TNG 72, which is a chemically mutated form of TNG 78, and TCS 17 to search for polymorphic genes residing on the intervals of QTLs conferring palatability and viscosity mapped identified in this study. Six starch synthesis genes, i.e., *PUL* (*Os04g0164900*), *SBE4* (*Os04g0409200*), *ISA2* (*Os05g0393700*), *GBSSI* (*Wx, Os06g0133000*), *SSII*-*3* (*Os06g0229800*), and *SSII*-*1* (*Os10g0437600*), and three polymorphic genes, i.e., *CPE* (COBRA putative expressed protein, *Os07g0604300*), *AGPs* (alpha-1,4-glucan-protein synthase, *Os07g0604800*), and *MADS18* (MADS-box family gene, *Os07g0605200*), resided in the QTL interval mapped to chromosome 7. The primer sequences and expected sizes of the amplicons, as determined by quantitative real-time (qPCR), are given in ESM Table S2.

RNA from TNG 78 and TCS 17 was extracted from approximately 20 mg of immature grains collected at 4, 7, 10, 15, 22, 29 days after pollination (DAP), respectively, during the second crop season of 2012 using the QIAGEN RNeasy Plant Mini Kit (Qiagen, Venlo, the Netherlands). The first-strand cDNA was synthesized with 1 μg total RNA using SuperScript III Reverse Transcriptase (Invitrogen, Life Technologies) in a volume of 20 μL, and qPCR was carried out using an ABI 7500 Sequence Detection System (ABI PRISM; Applied Biosystems, Foster City, CA) with the KAPA SYBR FAST qPCR kit –2× Master Mix Universal (Kapa Biosystems, Wilmington, MA). The relative expression of genes was calculated by the 2^−ΔΔCT^ method with *elongation factor 1*-*alpha* (*EFla*-*Q*) as the internal control (Livak and Schmittgen 2001). Relative expression in TNG 78 at 4 DAP was used as a standard for comparing the differential expression of genes between the two parents at the six stages of seed development.

## Results

### Variation in palatability and RVA profile parameters in the parents and RIL population

We evaluated eight physicochemical properties related to rice ECQ, including palatability and seven RVA parameters, in two parents and 190 RILs planted in two crop seasons. The palatability of cvs. TNG 78 and TCS 17 was the same in 2010–II, but could not be assessed for the other crop season because of insufficient rice grains for TCS 17. With the exception of PaT for 2010-II and PKV and PeT for 2011-I, all traits studied for the two crop seasons differed between cvs. TNG 78 and TCS 17 (ESM Table S3; ESM Fig. S1). The frequency distributions of all eight traits studied in the RIL population planted in the two crop seasons were continuous and showed transgressive segregation, indicating that all of these physicochemical properties were polygenic inherited traits. Nevertheless, HPV, BDV, CPV, and SBV showed a bimodal frequency distribution, implying that one major QTL might have made a major contribution to the phenotypic variation of these traits. Based on the paired difference *t* test analysis, PLS and six RVA parameters (the exception being PKV), differed significantly in the RIL population between the two different crop seasons (ESM Table S3), indicating that the viscosity of the cooked rice grains was affected by the environment.

### Correlation and distribution of palatability and RVA profile parameters

**Fig. 1:**
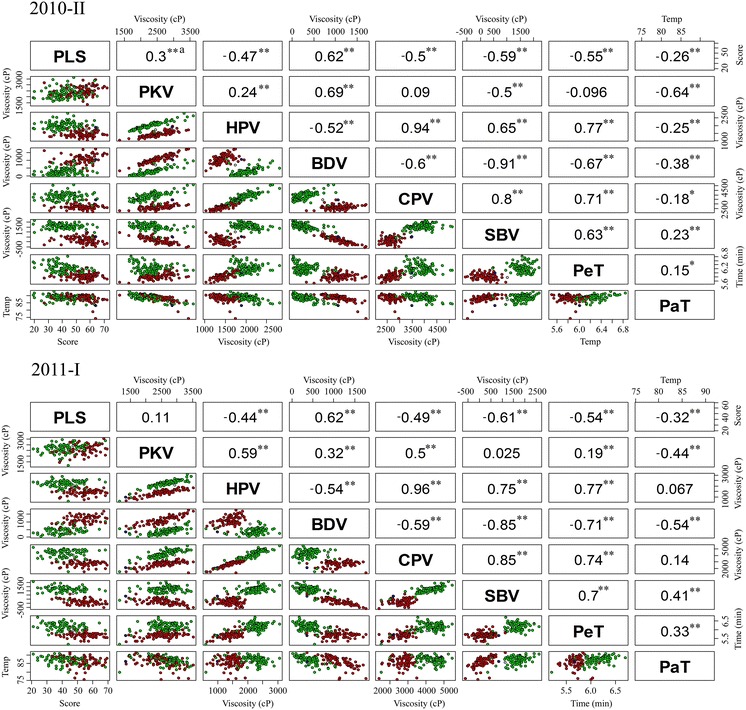
Spearman correlation coefficients and scatter plots of eight physicochemical properties of 190 recombinant inbred lines (RILs) measured in the second crop season of 2010 (*2010-II*) and the first crop season of 2011 (*2011-I*). Correlation coefficients significant at *p* < 0.05 and* p* < 0.01 by the Spearman’s rho test with two tails are indicated by* two asterisks* and* one asterisk*, respectively. Each RIL, genotyped by the derived cleaved amplified polymorphic sequences (dCAPs) functional marker of *Wx* (*Waxy*) locus, is indicated by *green*, *red*, or *black dots* as a homozygote to *Wx* ^*a*^ [allele of* Wx* encoding 20–30 % amylose content (AC)], a homozygote to *Wx* ^*b*^ (allele of* Wx* encoding 15–22 % AC), or a heterozygote, respectively.* PaT* Pasting temperature,* PKV* peak viscosity,* PeT* peak time,* HPV* hot paste viscosity,* BDV* breakdown viscosity,* CPV* cool paste viscosity,* SB*V setback viscosity. (Color figure online)

Pairwise correlation between palatability and the RVA parameters were generally consistent for both crop seasons. PLS was significantly positively correlated with BDV, but significantly negatively correlated with HPV, CPV, SBV, PeT, and PaT (Fig. 1). PLS was significantly correlated with PKV in 2010-II, but not in 2011–I. Five RVA parameters were positively or negatively correlated with each other; the exceptions were PKV and PaT, which did not show stable values, thus leading to inconsistent correlations with the other RVA parameters. HPV and CPV showed a high positive correlation (*r* = 0.94, 0.96), but BDV and SBV showed a high negative correlation (*r* = −0.91, −0.85) in both crop seasons.

Two-dimension scatter plots representing two different traits of physicochemical properties showed almost the same trends in 2010–I and 2011–II (Fig. 1). In addition, the distribution of one-pair traits of RILs agreed with correlation coefficients. For example, the correlation between HPV and CPV was highly positive, as shown by the linear relationship with a positive slope and by high correlation coefficients of >0.9; SBV and CPV showed a highly negative correlation, as seen by the linear relationship with a negative slope and by a high correlation coefficient of <−0.85. The dots shown on Fig. 1 were randomly distributed for PLS to PKV and HPV. PKV was positively correlated with HPV, BDV, and CPV, as seen by the positively inclined distribution, but negatively correlated with SBV, as indicated by the decreased distribution; these results were supported by the correlation coefficients.

To understand whether *indica* homozygotes with *Wx* ^*a*^ *Wx* ^*a*^ and *japonica* homozygotes with *Wx* ^*b*^ *Wx* ^*b*^ showed distinct physicochemical properties, we grouped the 190 RILs by genotype with the dCAPs marker designed on the basis of the FNP of *Wx*. From the dimension of PLS, most RILs with the *indica* genetic background and *japonica* genetic background overlapped (Fig. 1), which indicated that the *indica* and *japonica* rice grains had slightly different palatability. *Indica* and *japonica* homozygotes showed the same distribution of PKV and PaT, indicating no significant difference in PKV and PaT between *indica* and *japonica* rice. Values for HPV, CPV, SBV, and PeT were lower for *japonica* than *indica* homozygotes. However, values for only one RVA parameter, BDV, were lower for *indica* than *japonica* homozygotes (Fig. 1).

### QTLs conferring palatability and RVA parameters

**Fig. 2:**
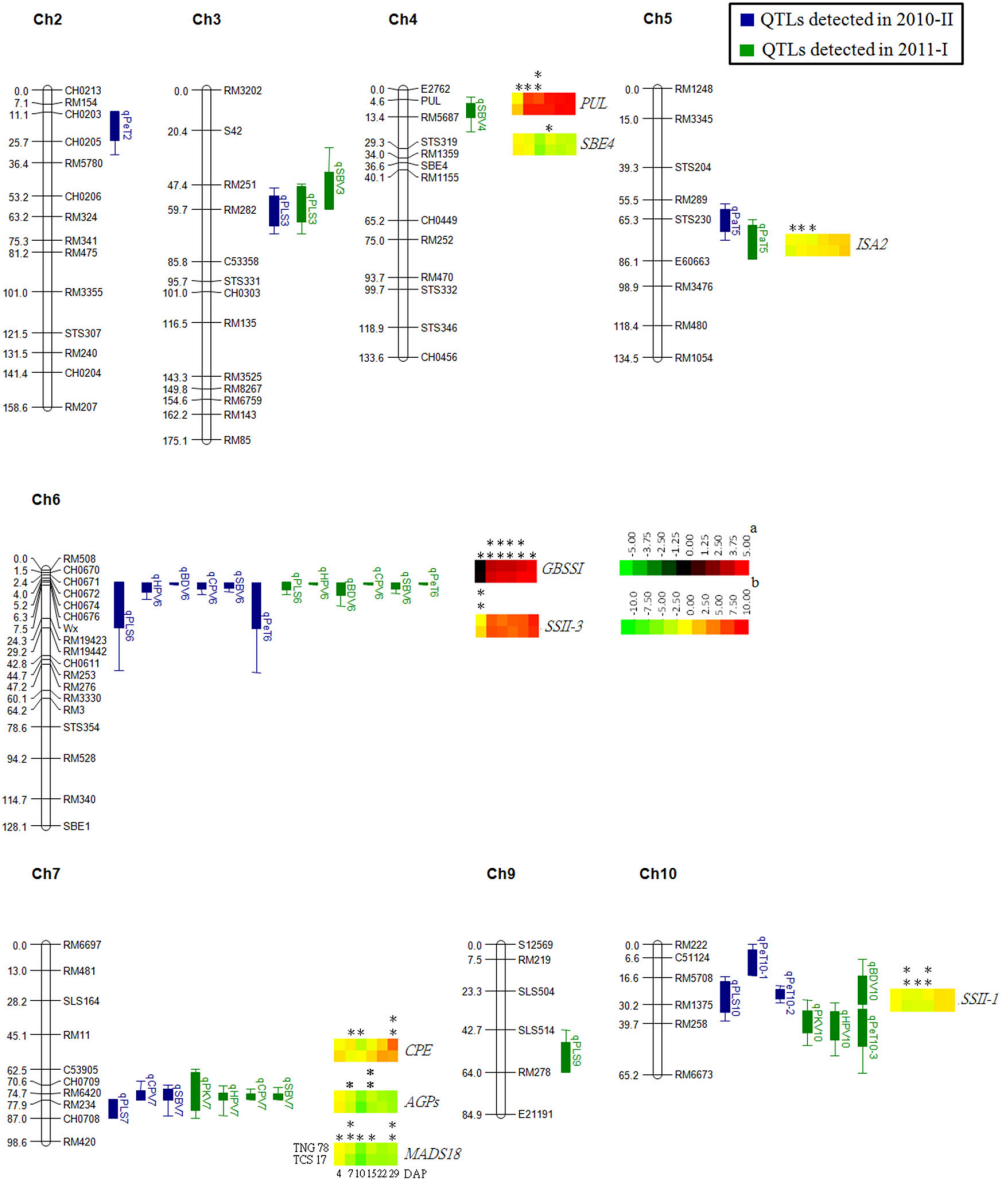
Interval maps of quantitative trait loci (QTLs) conferring eight physicochemical properties related to grain quality and gene expression of the candidate genes corresponding to the QTLs identified in this study. QTL intervals labeled with bars and lines extending out from the bar indicate 90 and 99 % likelihood, and QTLs identified in the second and first cropping seasons of 2010 and 2011 are labeled* blue* and* green*, respectively. The* heat maps* represent the gene expression of candidate genes residing in the identified QTL intervals analyzed in immature seeds harvested at 4, 7, 10, 15, 22, and 29 days after pollination subjected to real-time PCR.* Supercript a*,* b* Scales of heat maps for* granule-bound starch synthase-I* (*GBSSI*) and the other genes, respectively.* Two asterisks* and* one asterisk* *p* < 0.05 and 0.01, respectively, between the two parents, TNG 78 and TCS 17, as analyzed by *t* test. *PUL* pullulanase; *SBE4* starch branching enzyme genes; *ISA2* isoamylase genes 2; *SSII-3* starch synthesisII-3; *GBSSI* granule-bound starch synthase-I; *CPE* COBRA putative expressed protein; *AGPs* alpha-1,4-glucan-protein synthase; *MADS18* MADS-box family gene; *SSII-1* starch synthesisII-1. (Color figure online)

A total of 133 SSR and STS markers and six markers corresponding to starch synthesis genes were applied to the 190 RILs derived from TNG 78/TCS 17. These 139 molecular markers, spanning 96.1 % of the rice genome based on their physical mapping on the rice pseudomolecules established by the International Rice Genome Sequence Project (IRGSP), were used to construct a linkage map and detect QTLs for ECQ by interval mapping and marker regression analysis. The linkage map contained 12 LGs, covering 1,501.6 cM, with an average distance of 11.3 cM between two adjacent markers. The orders of most markers on the linkage map were consistent with the physical map according to the primer sequences of each marker (Fig. 2). Only eight LGs for which QTLs were detected, corresponding to eight chromosomes, are shown in Fig. 2; no QTLs were detected for the other LGs corresponding to chromosomes 1, 8, 11, and 12.

A total of 15 and 19 QTLs conferring palatability and the seven RVA parameters were identified in 2010–II and 2011–I, respectively. These 34 QTLs, explaining between 1.2 and 78.1 % of the phenotypic variation (PVE) were mapped on eight chromosomes in clusters. Ten pairs of QTLs were consistently detected in both seasons, but the remaining 14 QTLs were uncovered in one environment only (Table 1; Fig. 2).

**Table 1:** Quantitative trait locus parameters of eight physiochemical properties related to eating and cooking qualities

Traits^a^	QTLs	Chromosome	2010–II					2011–I				
			Position (cM)	Nearest marker	LOD	Effect^b^	PVE (%)	Position	Nearest marker	LOD	Effect	PVE (%)
PLS	*qPLS3*	3	59.81	RM282	4.66	2.90	5.9	58.41	RM282	3.58	2.83	6.4
	*qPLS6*	6	7.41	*Wx*	17.80	6.47	32.6	7.41	*Wx*	14.78	5.91	29.9
	*qPLS7*	7	81.91	RM234	3.40	2.59	5.2					
	*qPLS9*	9						58.71	RM278	3.65	3.11	8.5
	*qPLS10*	10	25.61	RM1374	4.02	2.90	6.6					
PKV	*qPKV7*	7						74.71	RM6420	3.51	−132.70	6.7
	*qPKV10*	10						39.21	RM258	4.66	144.33	9.7
HPV	*qHPV6*	6	7.41	*Wx*	39.06	−286.40	61.2	7.41	*Wx*	35.03	−403.49	58.3
	*qHPV7*	7						74.71	RM6420	4.77	−109.90	3.3
	*qHPV10*	10						39.21	RM258	3.36	87.08	2.8
BDV	*qBDV6*	6	7.41	*Wx*	43.19	377.93	67.4	7.41	*Wx*	43.68	359.34	69.0
	*qBDV10*	10						21.61	RM5708	3.01	71.26	2.7
CPV	*qCPV6*	6	7.41	*Wx*	50.47	−591.57	71.2	7.41	*Wx*	43.68	−775.63	62.3
	*qCPV7*	7	74.71	RM6420	3.29	−93.09	1.3	74.71	RM6420	3.01	−213.56	3.5
SBV	*qSBV3*	3						51.41	RM251	3.21	−99.83	1.4
	*qSBV4*	4						13.01	RM5687	5.08	−139.53	2.1
	*qSBV6*	6	7.41	*Wx*	59.40	−673.93	77.9	7.41	*Wx*	61.42	−728.51	78.1
	*qSBV7*	7	74.71	RM6420	3.72	−89.12	1.2	74.71	RM6420	4.15	−110.25	1.2
PeT	*qPeT2*	2	20.11	CH0205	3.39	−0.0558	4.1					
	*qPeT6*	6	7.41	*Wx*	33.52	−0.2116	56.8	7.41	*Wx*	33.44	−0.2437	64.1
	*qPeT10*-*1*	10	7.61	C51124	3.35	−0.0581	3.3					
	*qPeT10*-*2*	10	22.61	RM5708	7.46	−0.0859	9.6					
	*qPeT10*-*3*	10						39.21	RM258	3.63	0.0581	3.5
PaT	*qPaT5*	5	65.41	STS320	4.29	0.6475	8.8	77.41	E60663	3.97	0.9351	12.4

QTLs, Quantitative trait loci; LOD, limit of detection; PVE, phenotypic variation explained; 2010-II, second crop season of 2010, 2011-I, first crop season of 2011

^a^PLS, palatability; PKV, peak viscosity; HPV, hot paste viscosity; BDV, breakdown viscosity; CPV, cool paste viscosity; SBV, setback viscosity; PeT, peak time; PaT, pasting temperature

^b^Additive effect of alleles from *japonica* cv. Tainung 78 (TNG 78)

#### Palatability

Seven QTLs for PLS were detected in the two crop seasons, accounting for 5.2 to 32.6 % of PVE (Table 1; Fig. 2). Two QTLs, *qPLS3* and *qPLS6*, were detected in the two seasons and explained up to 6.4 and 32.6 % of phenotypic variation, respectively. The locus *qPLS6* was mapped adjacent to *Wx* and contributed considerably to variation in palatability—32.6 and 29.9 % in 2010–II and 2011–I, respectively. Two QTLs, *qPLS7* and *qPLS10*, were detected in 2010–II, and *qPLS9* was detected in 2011–I only. The TNG 78 alleles for all seven QTLs increased PLS.

#### Peak viscosity, hot paste viscosity, and breakdown viscosity

For PKV, two QTLs were detected in only one growing season, 2011–I, in which *qPKV7* and *qPKV10* contributed to 6.7 and 9.7 % of PVE, respectively. The TNG 78 allele of *qPKV7* decreased PKV, but that of *qPKV10* increased it (Table 1; Fig. 2).

One and three QTLs accounted for 2.8 to 61.2 % of the PVE for HPV in 2010–II and 2011–I, respectively (Table 1; Fig. 2). One QTL, *qHPV6*, was identified in both environments, explaining 61.2 and 58.3 % of the phenotypic variation, respectively. This major QTL was mapped around the *Wx* locus, with the largest effect; the TNG 78 allele decreased HPV. The other two QTLs, *qHPV7* and *qHPV10*, were detected in 2011–I only.

For BDV (derived from PKV − HPV), one major QTL, *qBDV6*, was detected in the two environments, and an additional QTL, *qBDV10*, was uncovered in 2011–I only (Table 1; Fig. 2). The major QTL, *qBDV6*, mapped nearby *Wx*, could explain 67.4 and 69.0 % of the PVE in both crop seasons; the TNG 78 allele increased BDV.

#### Cool paste viscosity and setback viscosity

Two QTLs for CPV, *qCPV6* and *qCPV7*, were consistently detected in the two crop seasons. The QTL *qCPV6* was mapped around *Wx* and contributed largely to phenotypic variance, accounting for up to 71.2 and 62.3 % PVE in the two environments; the TNG 78 allele decreased CPV (Table 1; Fig. 2).

For SBV (derived from CPV − PKV), we consistently detected two QTLs, *qSBV6* and *qSBV7*, in the two seasons, but an additional two QTLs*, qSBV3* and *qSBV4*, were uncovered in 2011–I only (Table 1; Fig. 2). The largest QTL, *qSBV6*, accounted for 77.9 % PVE and was mapped adjacent to *Wx*. The TNG 78 allele for all detected QTLs decreased SBV in both crop seasons.

#### Peak time and pasting temperature

A total of five QTLs accounted for PeT variation. Only one QTL, *qPeT6*, was consistently detected in the two seasons, and another three and one QTL were uncovered in 2010–II and 2011–I, respectively (Table 1; Fig. 2). The largest QTL, *qPeT6*, explained 56.8 and 64.1 % the phenotypic variation in the two crop seasons and was mapped adjacent to *Wx*; and the TNG 78 allele decreased PeT in both crop seasons.

Only one QTL for PaT was detected consistently in the two crop seasons. This locus, *qPaT5*, could explain 8.8 and 12.4 % of the PVE, respectively. The TNG 78 allele of *qPaT5* decreased PaT (Table 1; Fig. 2).

#### Co-localization of QTLs affecting physicochemical properties

To summarize the results of the interval mapping of QTLs affecting palatability and the seven RVA parameters, we found that the QTL clusters corresponding to the *Wx* locus simultaneously controlled PLS, HPV, BDV, CPV SBV, and PeT, but had no identified effect on PKV and PaT. The positive effects on phenotype in the QTL cluster corresponding to the *Wx* locus were from the TCS 17 allele for almost all traits except PLS and BDV; the other loci were clustered on chromosomes 3, 6, 7, and 10 across the two cropping seasons. For example, the QTL for PLS corresponding to the *Wx* locus on chromosome 6 coincided with the QTL for HPV, BDV, CPV, SBV, and PeT. The QTL for PLS near RM282 on chromosome 3 was also found for SBV. The QTL for PKV near RM6420 on chromosome 7 affected HPV, CPV, and SBV. The QTL for BDV near RM5708 on chromosome 10 was also found for PeT, and the QTL for PKV near RM258 on chromosome 10 was also found for HPV and PeT. Therefore, we suggest that pleiotropic effects or closely linked genes influence the starch physicochemical properties of cooked rice grains.

### Verification of genetic factors conferring palatability and viscosity of cooked rice grains through RIL

As the starch composition of the rice grain contributes markedly to the physicochemical properties of the cooked rice grain, we examined nine genes involved in starch biosynthesis (Yan et al. 2011) to determine their correlation with palatability and the studied RVA parameters. Six of the nine genes showed allelic variation in cvs. TNG 78 and TCS 17 (ESM Table S1). The genotypes of these six polymorphic genes, *Wx* (KF984387 for TNG 78, KF984392 for TCS 17), *PUL* (KF984389 for TNG 78, KF984394 for TCS 17), *SBE4* (KJ008711 for TNG 78, KJ008712 for TCS 17), *SBE1* (KF984385 for TNG 78, KF984390 for TCS 17), *ISA1* (KF984388 for TNG 78, KF984393 for TCS 17), and *SSII*-*1* (KF984386 for TNG 78, KF984391 for TCS 17), in the RIL population along with phenotype data of palatability and RVA profile parameters were analyzed by marker regression analysis. *Wx*, encoding GBSSI, which regulates the ratio of AC and amylopectin, contributed considerably to most of the traits, except PKV in 2011–I and PaT in both crop seasons (Table 2). The PVE of *Wx* and the effect of the *Wx* ^*b*^ allele from TNG 78 on PLS and RVA parameters were similar to those estimated by interval mapping (Tables 1, 2). *PUL* was the second major genetic factor influencing RVA parameters, having a noteworthy effect on HPV, CPV and SBV in the two crop seasons and on PLS, PKV, BDV, and PaT in one crop season only. The TNG 78 allele of *PUL* increased the positive effect of most of these traits, except for PKV and BDV. *SBE4* influenced PKV and BDV in both seasons and HPV, CPV, SBV, and PaT in 2010–II only (Table 2), and the TNG 78 allele of *SBE4* increased SBV and PaT. *SBEI* was detected as significantly contributing to PLS in 2010–II only, when the TNG 78 allele of *SBE1* increased PLS. The other gene, *SSII*-*1*, did not have a significant association with any trait.

**Table 2:** Significant genetic factors contributing to the palatability and viscosity of cooked rice grains

Traits^a^	Significant genetic factors^b^																	
	*Wx*		*PUL*		*SBE4*		*SBE1*		*ISA1*		*Wx*:*PUL*		*Wx*:*SBE4*		*PUL*:*SBE4*		*SBE1*:*ISA1*	
	Effect^c^	PVE	Effect	PVE	Effect	PVE	Effect	PVE	Effect	PVE	Effect	PVE	Effect	PVE	Effect	PVE	Effect	PVE
PLS	−6.50	32.9					−0.87	3.4	−0.48	3.9							−4.14	3.0
	−6.37	35.1	1.69	2.4														
PKV	−81.41	4.2	−36.00	5.5	63.04	7.4									191.12	4.8		
					94.97	4.3												
HPV	298.18	66.1	−12.94	1.6	37.36	1.9					57.48	0.6			77.62	0.9		
	400.71	57.8	60.95	1.3														
BDV	−379.56	70.8	−30.30	1.7	30.66	2.0			31.35	0.5			−58.38	0.4	115.36	1.3		
	−352.33	68.9			43.12	1.4							−61.40	0.5				
CPV	596.11	74.7	23.70	0.8	54.46	0.5					112.82	0.6						
	777.52	64.8	132.46	1.9														
									−55.21	0.5								
SBV	678.16	80.9	64.07	1.7	−9.95	0.7					91.50	0.4			−135.04	0.6		
	723.24	80.6	56.23	1.4							141.86	0.7						
PeT	0.20	53.4																
	0.23	58.1																
PaT			0.15	3.7	−0.25	5.1									−0.70	3.1		

^a^The effects and PVE of each genetic factor obtained for each trait measured in the crop seasons 2010–II and 2011–I are indicated as upper and lower rows, respectively

^b^All genetic factors contributed to the phenotypic variance at a significance level of* p* < 0.05

^c^The additive effect of an allele from TNG 78 for each single gene. For a two-gene interaction, the effects were estimated as [(JJ + II) − (JI + IJ)]/2 for which JJ and II indicate the RILs possessing two genes which were homozygous to TNG 78 or TCS 17, and JI and IJ indicate the RILs possessing one gene homozygous to TNG 78 and the other gene homozygous to TCS 17, and vice-versa

**Fig. 3:**
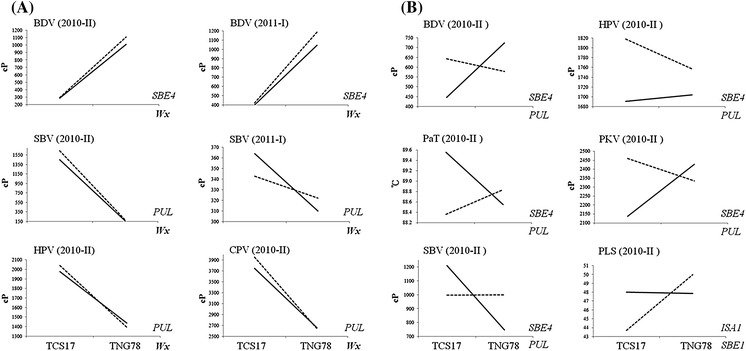
The plots of two-way gene interaction. **a** *Wx* interacting with *SBE4* and *PUL* influences BDV, SBV, HPV, and CPV. **b** Interaction of PUL with *SBE4* and *SBE1* with *ISA1* played a significant role in BDV, HPV, PaT, SBV, CPV, and PLS. The genotypes homozygous in TCS 17 or TNG 78 are indicated for the genes along the* x-axis*; the other interacting genes are indicated by *dashed* and *solid lines* as homozygous in TCS 17 and TNG 78, respectively. *SBE4* starch branching enzyme genes 4; *PUL* pullulanase; *ISA1* isoamylase genes 1

The biochemical pathway of starch synthesis is a complicated network of gene regulation and interaction of SSRGs. Gene interaction at two-gene level and revealed that *Wx*:*PUL*, *Wx*:*SBE4*, *PUL*:*SBE4*, and *SBE1*:*ISA1* played significant roles in physicochemical properties of cooked rice grains (Table 2; Fig. 3). The interaction of *Wx*:*PUL* had impact on HPV, CPV, and SBV, in which interaction of two genes of same genotypes for which two genes were homozygous to TNG 78 and TCS 17 contributed larger effects than that of two genes of opposite genotypes for which one gene homozygous to TNG 78 and the other was homozygous to TCS 17, vice versa. Nevertheless, the interaction of opposite genotypes of *Wx*:*SBE4* and *SBE1*:*ISA1* reduced BDV and PLS, respectively. The interaction of *PUL*:*SBE4* could not be neglected for its influences on PKV, HPV, BDV, SBV, and PaT, and the interaction of opposite genotypes of *PUL*:*SBE4* decreased SBV and PaT (Table 2; Fig. 3).

*Wx* is a primary gene regulating several physicochemical properties of the cooked rice grains, as indicated by CIM and marker regression analyses (Tables 1, 2). The major impact of *Wx* on the phenotypic variation of all traits in the RIL population might influence the contribution of the other genes. We observed that different alleles of *SBE4* contributed differently to BDV obtained from the two crop seasons in a genetic background of *Wx* ^*b*^ implied by homozygotes of TNG 78, but not of *Wx* ^*a*^ implied by homozygotes of TCS 17 (Fig. 3). On the other hand, different *PUL* alleles exhibited different effects on SBV and CPV in 2010–II in the genetic background of *Wx* ^*a*^. However, there was no obvious dependence of genetic background for the other gene interaction. These genes were expressed and interacted with each other in a specific genetic background in the *Wx* ^*a*^ and/or *Wx* ^*b*^ genotypes in a specific environment (Table 2; Fig. 3).

### Expression of candidate genes responsible for palatability and RVA parameters

We analyzed the expression of those starch synthesis genes which contributed to palatability and seven RVA parameters, resided in the intervals of QTLs, and exhibited allelic variation between two parents, TNG 78 and TCS 17, in immature caryopsis during the grain-filling stages (Table 1; Fig. 2). The aim of this analysis was to determine whether these genes were responsible for grain starch viscosity. We quantified the relative expression of these six genes at six grain-filling stages; the genes were *PUL* encoding pullulanase; *SBE4* encoding starch branching enzyme; *ISA2* encoding isoamylase-type starch-debranching enzyme 2 and 1, respectively; *GBSSI* encoding granule-bound starch synthase-I; *SSII*-*3* encoding starch synthase II-3; *SSII*-*1* encoding starch synthase II-1 (ESM Table S2). These six genes showed differential gene expression between the two parents, which might account for the different physicochemical properties conferring the ECQ of *japonica* rice TNG 78 and *indica* rice TCS 17 and the phenotypic variation in physicochemical properties of rice grain starch in the RIL population.

*GBSSI*, located on chromosome 6 and corresponding to *Wx*, was expressed at considerably higher levels than the five other starch synthesis genes during all six grain-filling stages. Moreover, the expression of *GBSSI* differed greatly between TNG 78 and TCS 17, with a two to 25-fold higher expression in TCS 17. This higher expression might explain the phenotypic variation of PLS, HPV, BDV, CPV, SBV, and PeT, corresponding to the major QTL clusters of these traits (Fig. 2). In the same interval of several QTLs corresponding to the above phenotypes, *SSII*-*3* near *GBSSI* showed distinct expression at 4 DAP. The gene expression of *SSII*-*1* also differed between TNG 78 and TCS 17, with significant levels at 7, 10, and 15 DAP. *SSII*-*1* is located in the interval between RM5708 and RM258 corresponding to the QTL cluster of *qPLS10*, *qPeT10*-*2*, *qPKV10*, *qHPV10*, *qBDV10*, and *qPeT10*-*3*. The higher expression of *SSII*-*1* in TNG 78 than TCS 17 might account for the phenotypic variance of PLS, BDV, CPV, SBV, PeT, and PaT in a *japonica* and *indica* background, respectively (Table 2). The gene expression of *PUL*, residing in the interval of *qSBV4*, differed between TNG 78 and TCS 17 in the early grain-filling stages (<10 DAP) and was one of the factors resulting in variation in SBV in the RIL population and variation in CPV and SBV in *japonica* homozygotes (Table 2). Another gene located in the interval of *qSBV4* was *SBE4*, which was also expressed differently between the parents, with a significance difference at 15 DAP. *ISA2*, residing in the interval of *qPaT5*, showed differential gene expression levels in the early grain-filling stages, with higher expression in TNG 78 at 4 DAP and lower at 7 and 10 DAP compared with that in TCS 17 (Fig. 2).

## Discussion

Two rice subspecies, *indica* and *japonica*, showed different physicochemical properties in terms of grain quality. In terms of country and ecogeographical differences, lowland *indica* tends to form discrete and non-sticky grains when cooked and is grown throughout tropical Asia; *japonica* has moderate elasticity and stickiness and is typically found in temperate East Asia, upland areas of Southeast Asia, and high elevations in South Asia (Kang et al. 2006). Consumers in Taiwan, similar to those in Japan, Korea, and Northern China, prefer *japonica* because of the sticky, gelatinous, and soft texture of the rice grain; however, most consumers in the world prefer *indica*. The physicochemical properties of rice grains, which depend intrinsically on the ratio of amylose and amylopectin in the starch, are the major components affecting the ECQ of cooked rice grains. However, these physicochemical properties also determine their suitability for particular end uses; for example, low GT is preferred in the manufacturing of rice bread and beer. In Taiwan, *japonica* rice is the daily staple food, and *indica* rice has distinct applications in the manufacturing of rice noodles, rice cakes, and other derived products. We selected two varieties, *japonica* cv. Tainung 78 (TNG 78) and *indica* cv. Taichung Sen 17 (TCS 17) as mapping parents to reveal QTLs conferring physicochemical properties based on their primary different breeding goals. TNG 78 was selected for its good grain quality, including fragrance, giant embryo, low AC, and soft GC. TCS 17, in addition to its known high yield and resistance to blast and brown planthopper, has a considerably high AC level and has been extensively used in manufacturing rice-derived products.

The physiochemical properties of rice grain are complicated and influenced by both genes and environment. Two alleles of the *Wx* locus, *Wx* ^*a*^ and *Wx* ^*b*^, have undergone diversified selection in *indica* and *japonica*: the *Wx* ^*a*^ allele is predominant in *indica* rice and *Wx* ^*b*^ in *japonica* rice, thus resulting in different AC in rice grains and, consequently in different physicochemical properties (Wanchana et al. 2003). Even the genotypes with the same allele of *Wx* ^*a*^ or *Wx* ^*b*^ have various textures, indicating that other genes are involved in determining rice ECQ. Nevertheless, the effects of other starch synthesis genes and starch synthesis-regulated genes expressing and interacting in specific genetic backgrounds cannot be neglected in examining the physicochemical properties of the starch in rice grains (Liu et al. 2009; Sun et al. 2011; Tian et al. 2009). Regarding our two parent varieties, most of the physicochemical properties of TNG 78 differed from those of TCS 17, and 190 RILs of TNG 78 × TCS 17 showed transgressive segregation with continuous distribution in two crop seasons, 2010–II and 2011–I (ESM Table S3, ESM Fig. S1). A total of 34 QTLs, with a range in PVE between 1.2 and 78.1 %, were identified in the two crop seasons (Table 1; Fig. 2). Several viscosity traits exhibited a bimodal distribution (ESM Fig. S1), which indicated a major gene was involved in regulating these traits. We found a major QTL cluster consisting of *qPLS6*, *qHPV6*, *qBDV6*, *qSBV6*, and *qPeT6* around *Wx* on chromosome 6 that was detected consistently in the two crop seasons at significant levels (Table 1; Fig. 2). *Wx* was also a major contributor to the observed phenotypic variation of these six traits, with the PVE ranging from 29.85 % for PLS in 2011–I to 71.2 % for BDV in 2010–II. The *Wx* ^*a*^ and *Wx* ^*b*^ homozygotes of the 190 RILs formed a distinct group for HPV, BDV, CPV, SBV, and PeT, with slight differences for PLS, but not for PKV and PaT, on the scatter plot (Fig. 1). In addition, at least seven loci located on the other seven chromosomes contributed to the observed phenotypic variation in physicochemical properties, ranging from 1.2 % for *qSBV7* to 12.4 % for *qPaT5* (Table 1; Fig. 2). We also detected two-gene interactions contributing to the phenotypic variation in the physicochemical properties (Table 2; Fig. 3). All physicochemical properties, except for PKV, differed significantly between crop seasons (ESM Table S3). These results indicate that the viscosity of the cooked rice grains, as reflected by palatability and RVA parameters, was polygenic inherited with gene interaction.

PLS and six RVA parameters—the exception being PKV—differed significantly in the two seasons (ESM Table S3), and 14 of 34 QTLs were uncovered in only one environment (Table 1; Fig. 2). Our results are consistent with those of previous studies which reported that only *Wx* was detected for five RVA parameters in the two environments (Bao et al. 2000) and that various QTLs were detected for different viscosity parameters in the two seasons (Wang et al. 2007). In addition to these genes, environment also has a great impact on grain quality. Temperature, specifically in the grain-filling stages, is known to be an important factor influencing grain quality (Lur et al. 2009; Yamakawa et al. 2007; Zhao and Fitzgerald 2013). Lur et al. (2009) reported that the average temperature at 15 days after heading was negatively correlated with AC, PKV, and BDV. We observed a variation in heading date, within a range of approximately 1 month, among the two parents and 190 RILs cultivated in the two crop seasons. In addition, in Taiwan, the average daily air temperature decreased during the second crop season and increased during the first crop season, leading to a higher grain quality in the crop harvested in the second crop season compared to that of the first crop season(Lur et al. 2009). The variation in heading date associated with variation temperature was attributed to the physiochemical properties obtained and some of the QTLs detected from two different crop seasons (Table 1; Fig. 2; ESM Table S3; ESM Fig. S1).

Allelic variation among different germplasms might account for the variation in physicochemical properties. Thus, uncovering specific alleles responsible for a particular characteristic or for viscosity parameters would be helpful when breeding for specific goals, such as the long-term selection of *japonica* and *indica* rice for different end-uses. We found that the major QTL cluster corresponding to the *Wx* locus was simultaneously responsible for PLS, HPV, BDV, CPV SBV, and PeT in the 190 RILs of TNG 78/TCS 17 (Table 1; Fig. 2). The *Wx* locus has also been found to affect most of the viscosity parameters, with the exception of PKV, in the RILs of *indica* Zhenshan 97/*indica* Delong 208 and five RVA parameters (HPV, CPV, BDV, CSV, and SBV) in the DH of *indica* Zai-Ye-Qing 8/*japonica* Jing-Xi 17 in two environments (Bao et al. 2000; Wang et al. 2007). In addition, the *Wx* locus was reported to greatly affect AC and GC, with minor effects on alkali spreading in the RIL population of Zhenshan 97 and Delong 208 (Wang et al. 2007). Therefore, we suggest that *Wx* plays an important role in the control of RVA parameters in *indica* and *japonica* rice. Nevertheless, we observed phenotypic variation in physicochemical properties in the same group with the same *Wx* allele, namely, homozygotes of *Wx* ^*a*^ or *Wx* ^*b*^, on scatter plots (Fig. 1), suggesting the participation of other starch synthesis genes or SSRGs (Bao et al. 2006b; Yan et al. 2011).

In this study, the second QTL cluster correlated with PLS, CPV, SBV, PKV, and HPV and was mapped near RM6420 on chromosome 7 (Table 1). This QTL was also a major contributor to phenotypic variation in these traits, with the PVE ranging from 1.2 % for SBV in 2010–II to 6.7 % for PKV in 2011–I. Based on the physical map of the markers used in QTL mapping, we found that only one QTL conferred grain quality; this QTL has been found to be significantly responsible for PKV, pasting temperature (PaT), pasting time (PeT), and time needed from gelatinization to peak (BAtime) (Wang et al. 2007). A QTL, *qPGWC*-*7*, controlling percentage of grain with chalkiness (PGWC), has also been mapped to the same chromosome segment (Zhou et al. 2009). Therefore, an important gene residing in the interval containing the QTL cluster on chromosome 7 appears to influence rice grain quality and appearance by affecting the physicochemical properties. Nevertheless, the other QTLs conferring palatability and the RVA parameters we uncovered did not correspond to previous findings, and our newly discovered QTLs for the ECQ provide new insights into the development of rice grain quality. In particular, one QTL cluster consisting of *qPLS10*, *qPKV10*, *qHPV10*, *qBDV10*, *qPeT10*-*2*, and *qPeT10*-*3* was mapped between RM5708 and RM258 on chromosome 10, and *SSII*-*1*, involved in synthesizing amylopectin, resided near RM1357 in the interval (Table 1; Fig. 2).

The physicochemical properties of the cooked rice grain can be reflected by palatability and viscosity profiles as revealed by using Toyo Test Meter and RVA profiles and can be considered in evaluating rice ECQ, respectively (Bao and Xia 1999; Deffenbaugh and Walker 1989; Lestari et al. 2009; Sun et al. 2011). Sun et al. (2011) found that palatability was negatively correlated with protein, but not pasting properties, RVA parameters, AAC, or alkali digestion value in 8 *japonica* rice varieties). However, in our study, with the exception of PKV in 2011–I, palatability was correlated with RVA parameters, with PLS positively correlated with PKV and BDV, but negatively with the other variables (Fig. 1). The likely reason for this discrepancy with the results of Sun et al. (2011) might be the different germplasm used: eight *japonica* varieties versus 190 RILs of *japonica* TNG 78/*indica* TCS 17. In their overall sensory evaluation of 40 *japonica* varieties, Wu et al. (2007b) found a partially negative correlation with PeT, but a positive correlation with HPV. PKV and BDV were positively correlated with gel consistency, but negatively correlated with AC, HPV, CPV, and SBV. In addition, low PaT and PeT, corresponding to high alkaline spreading, have been suggested to contribute to good cooking quality (Jia et al. 2008). Sun et al. (2011) also reported that AC and alkali digestion values were significantly correlated with RVA pasting properties, such as BDV, SBV, consistency viscosity, and PeT. Therefore, to meet consumers’ preference for rice grains with a gelatinous and elastic texture, the breeding goal for selecting good ECQ should be low AC associated with high PLS, PKV, and BDV and low HPV, CPV, SBV, PaT, and PeT. From the scatter plots for the 190 RILs which were genotyped with the FNP of *Wx* to assign *japonica* or *indica* homozygotes, RILs homozygous for TNG 78 tended to show lower HPV, CPV, SBV, and PeT values, but higher BDV than did the RILs homozygous for TCS 17 (Fig. 1). In addition, the interval mapping of ECQ QTLs revealed that the alleles for TNG 78 conferred positive effects on PLS, BDV and PaT, but negative effects on HPV, CPV, SBV, and PeT (Table 1). These findings are consistent with the expectation that *japonica* usually has a soft texture and that TNG 78 can be considered a good germplasm to promote rice grain quality in breeding programs.

Because starch constitutes approximately 90 % of rice grain and is a major main component determining the physicochemical properties of the cooked rice grain, starch synthesis genes and other regulatory genes are considered to be the most important genetic factors influencing rice grain quality. An association study of 118 glutinous rice accessions of eight *japonica* varieties revealed that ten of 17 SSRGs regulated RVA profile parameters (Yan et al. 2011) and that five SSRGs differentiated eating quality (Sun et al. 2011). In our study, we used six of the polymorphic SSRGs identified by Yan et al. (2011) and subjected these to linkage and marker regression analyses. We found that *PUL*, *SBE4*, *GBSSI*, and *SSII*-*1* resided in the intervals of the identified QTLs conferring palatability and RVA parameters and subsequently analyzed the gene expression of these four genes during grain-filling stages (Table 2; Fig. 2). We also analyzed the expression of two polymorphic genes, *ISA2* and *SSII*-*3,* located in the QTL intervals on chromosomes 5 and 6, making a total of six SSRGs which we considered to be candidate genes controling the ECQ of cooked rice grains. These six genes showed differential gene expression between the two parents during some or all of the grain-filling stages (Fig. 2; ESM Table S2). The *Wx* locus corresponded to the major QTL cluster conferring PLS, HPV, BDV, CPV SBV, and PeT, but not PKV and PaT, and contributed considerably to most traits in at least one crop season (Tables 1, 2; Fig. 2). *Wx*, encoding GBSSI, a key enzyme regulating the ratio of amylose content and amylopectin, dominates as a determinant of the physicochemical properties of starch; different alleles with full, leaky, and null function resulted in dissimilar grain appearance and ECQ because of varied AC and starch-granule formation and arrangements (Liu et al. 2009; Tian et al. 2009; Zhang et al. 2012). *Wx* ^*a*^ and *Wx* ^*b*^ are predominant in *indica* and *japonica*, with diversified selection, and therefore leads to distinct differences in AC in *indica* and *japonica* because *Wx* ^*b*^, as a result of its partial function due to an single nucleotide polymorphism, leading to cryptic splicing, reduces the AC level in *japonica* rice (Yamanaka et al. 2004). Moreover, the expression of *GBSSI* was significantly higher in *indica* TCS 17 than in *japonica* TNG 78 during all grain-filling stages (Fig. 2). We therefore conclude that the low AC level synthesized in RILs homozygous to TNG 78 caused low HPV, CPV, SBV, and PeT levels, but high PLS and BDV levels (Table 1; Fig. 1).

*SSII*-*1*, also named *SSIIc*, is the SSRG gene responsible for amylopectin chain elongation; it is expressed in the early grain-filling stage (Hirose and Terao 2004). In this study, *SSII*-*1* was found to reside in the interval containing the QTL cluster of *qPLS10*, *qPKV10*, *qHPV10*, *qBDV10*, *qPeT10*-*2*, *qPeT10*-*3*, and its expression significantly differed in the two parents during early grain-filling stages (Tables 1, 2; Fig. 2). Another starch synthesis gene, *SSII*-*3*, also named *SSIIa*, which was found to reside in the interval containing the major QTL cluster on chromosome 6 and near *GBSSI*, showed differential expression in the two parents at 4 DAP (Fig. 2). The contribution of *SSII*-*3* to the ECQ is considerable as the SSII-3 enzyme initiates short-chain elongation of amylopectin and corresponds to *Alk*, as shown by its deficiency resulting in altered physicochemical properties and gelatinization temperature (Bao et al. 2006a; Gao et al. 2003; Umemoto et al. 2002; Zhang et al. 2011). Our data support previous results showing that SS genes regulating the synthesis and structure of amylopectin also influence the starch physicochemical properties of waxy and non-waxy rice grains (Bao et al. 2006a; Gao et al. 2003; Sun et al. 2011; Umemoto et al. 2002; Yan et al. 2011; Zhang et al. 2011).

*PUL*, which is localized in the interval of *qSBV4*, contributed to palatability and the seven RVA parameters either on its own or by interacting with *Wx* and *SBE4* (Tables 1, 2; Figs. 2, 3). The gene expression of *PUL* differed in the two parents at early grain-filling stages (Fig. 2). *PUL* showed strong associations with PKV, HPV, CPV, BDV, PeT, and PaT in 118 glutinous rice accessions and with AC in 33 *indica* and 37 *japonica* accessions in an association study, but not in the RILs of *indica* Zhenshan 97/*indica* Delong 208 and the DH of *indica* Zai-Ye-Qing 8/*japonica* Jing-Xi 17 by interval mapping (Bao et al. 2000; Tian et al. 2009; Wang et al. 2007; Yan et al. 2011). One possible reason for *PUL* not being responsible for the ECQ in the interval mapping of the two QTL clusters is the lack of polymorphism between the two mapping parents; a second, more likely reason was masked by *Wx* because only one QTL of SBV was detected by interval mapping (Table 1). Nevertheless, Yan et al. (2011) found that *PUL* may be a critical gene determining RVA parameters in glutinous rice, which was expected in the *wx* genetic background. In our study, *PUL* contributed to seven traits associated with physicochemical properties, with the exception of PeT, as evidenced by marker regression analysis, and exhibited different effects on SBV and CPV specifically in the *indica* genetic background (Table 2; Fig. 3). The other starch-debranching enzyme is ISA, which can debranch amylopectin and glycogen. *ISA* was found to control GC and GT in an association study (Tian et al. 2009). We found that two of three *ISA* isoforms contributed to the physicochemical properties of cooked rice grains: *ISA1* itself or when interacting with *SBE1* affected PLS, BDV, and SBV, and *ISA2* in the interval of *qPaT5* was expressed differently in the two parents during early grain-filling stages (Table 2; Figs. 2, 3). *SBE4* did not correspond to any QTL by interval mapping, but it did influence the six physicochemical properties by mainly interacting with *Wx* and *PUL* (Table 2), which implies that it has little impact on grain quality; this result is in accordance with the discovery that *SBE* had minor effects on AC, GC, and palatability (Tian et al. 2009; Sun et al. 2011). The results of our study and of previous studies suggest that physicochemical properties of cooked rice grains are regulated primarily by *GBSSI*—largely by *PUL* and *SBE4* and less by *SSII*-*3*, *ISA1*, and *ISA2*.

Our study of gene interaction at the two-gene level revealed that *Wx* interacting with *PUL* and *SBE4*, *PUL* interacting with *Wx* and *SBE4*, and *SBE1* interacting with *ISA1* significantly contributed to several physiochemical properties. Specifically, different alleles of *SBE4* and *PUL* uncovered various physicochemical properties in a given genetic background of TNG 78 and TCS 17, respectively (Fig. 3). Consequently, modification of a single gene in rice breeding programs is inadequate because other minor SSRGs and gene interaction may play additional roles in regulating the physicochemical properties of rice starch.

None of the known starch synthesis genes or SSRGs resided in the interval containing the QTL cluster of *qPLS7*, *qCPV7*, *qSBV7*, *qPKV7*, and *qHPV7*, which was mapped near RM6420 on chromosome 7 (Table 1). Interestingly, one QTL, *qPGWC*-*7*, which controls PGWC, was also detected in the same region by comparative physical mapping based on the primer sequences of flanking markers on Rice Pseudomolecules (http://rice.plantbiology.msu.edu/) (Zhou et al. 2009). After delineating the overlapping chromosome segment, only three genes—*CPE* encoding COBRA-like protein, *AGPs* encoding a protein similar to UDP-glucose protein transglucosylase 1 (UPTG1), and *MADS18* encoding MADS box protein—were polymorphic between TNG 78 and TCS 17. Three polymorphic genes exhibited different gene expression profiles during grain-filling in TNG 78 and TCS 17 (Fig. 2). Even *MADS18* was significantly upregulated in TNG78 during grain-filling (Fig. 2); however, it might not contribute to grain quality because *OsMADS18* phylogenetically clustered with the *SQUA/Apetala1* (*AP1*) group and is widely expressed in all plant tissues (Arora et al. 2007; Fornara et al. 2004). *CPE* was found to be significantly upregulated at 7 and 29 DAP, but downregulated in TNG 78 during primary and late or medium grain-filling. *CPE*, which encodes COBRA-like protein, belongs to *Oryza sativa brittle culm 1 like 6* (*OsBC1L6*) and is primarily expressed in endosperm at various stages; it is required for the development of endosperm (Dai et al. 2009; Hochholdinger et al. 2008). We found that *AGPs* was significantly upregulated in TNG 78 at 7 and 15 DAP, which might lead to increased enzymatic activities to accumulate ADP-glucose; indeed, *AGPs* encoding *α*-1,4-glucan-protein synthase (UDP-forming) plays an important role in the synthesis of cellulose (Glaser 1985). Several cellulose synthase genes were found to be up- or downregulated in CSSL50-1, characterized as having high grain chalkiness, starch content, AC, sucrose content and protein content, but low in SBV, HPV, CPV, and consistent viscosity. Compared to its near-isogenic line CSSL50, CSSL50-1 showed reduced cellulose synthesis and increased hemicellulose hydrolysis, possibly due to enhanced sucrose and starch synthesis at the cost of cell-wall-related non-storage polysaccharides, which resulted in the altered starch composition and physicochemical properties of CSSL50-1 (Liu et al. 2010). *CPE*, which is involved in endosperm development, and *AGPs*, which influences carbon partition in endosperm during rice grain-filling stages, are two candidate genes for the QTL cluster on chromosome 7, which is responsible for grain appearance and physicochemical properties.

Five SSRGs, *PUL*, *SBE4*, *Wx*, *SBE1*, and *ISA1*, contributed to the variation in palatability and seven RVA parameters either individually or through interaction with the other SSRGs in one or both crop seasons (Table 2). From our analysis of the 190 RILs planted in the two environments, *Wx* and *PUL*, the major genes, were mostly associated with seven traits, *SBE4* contributed to six traits in only one or both environments; *SBE1* and *ISA1* were associated with one and three traits, respectively, in one crop season (Table 2). The alleles of *Wx*, *PUL*, and *SBE4* from TNG 78 promoted good ECQ, as evidenced by their effect of increasing PLS, PKV, and BDV, and reducing HPV, CPV, SBV, PeT, and PaT, with an exception of *PUL* on PLS (Table 2). Grain quality, reflected by physicochemical properties, is controlled by genes and the environment, whereby gene expression is regulated by other genes depending on the genetic background. Modern rice cultivars are designated as *indica* or *japonica*, with *Wx* alleles fixed as *Wx* ^*a*^ or *Wx* ^*b*^. How to fine-tune the grain quality of Mega varieties without *Wx* is important for breeding high-quality rice cultivars. Our study provides insight into a breeding strategy in which the improvement of grain quality could focus on *SBE4* for *japonica* rice and *PUL* for *indica* rice. In addition, specific gene interactions in a given genetic background cannot be neglected because of the complicated network of gene regulation in starch synthesis and its consequences on grain quality (Pandey et al. 2012; Tian et al. 2009).

Consumers are increasingly demanding good rice ECQ and, consequently, good ECQ has become a priority goal of rice breeding programs. TNG 78, which possesses superior characters of grain quality, is a useful genetic germplasm and can be used in rice breeding programs. The availability of the QTLs with closely linked markers we uncovered will provide efficiency and precision in marker-assisted selection. The gene expression of *PUL*, *ISA2*, *GBSSI*, and *SSII*-*3* was higher in TCS 17 than TNG 78 and positively correlated with phenotype because TCS 17 showed higher AC, HPV, CPV, SBV, PeT, and PaT. The gene expression of *SBE4* was higher in TNG 78 than in TCS 17 and negatively correlated with CPV, SBV, and HPV. Our studies provide valuable information that will help improve rice ECQ through the use of different SSRGs for breeding *indica* or *japonica*—*SBE4* for *japonica* and *PUL* for *indica*. The closely linked markers from stable expressed QTLs in two crop seasons and functional markers of SSRGs we have identified can facilitate the development of new rice varieties with the desired grain quality by marker-assisted selection.

## Electronic supplementary material

Below is the link to the electronic supplementary material.

Supplementary material 1 (PDF 658 kb)
