# Supplementary material for: Genetic factors responsible for eating and cooking qualities of rice grains in a recombinant inbred population of an inter-subspecific cross
Source: Mol Breed. 2014 Apr 4;34(2):655–73. doi: 10.1007/s11032-014-0065-8 (PMC4092229; doi:10.1007/s11032-014-0065-8)
Supplement: Supplementary file 1 — Supplementary material 1 (PDF 658 kb) [file 11032_2014_65_MOESM1_ESM.pdf]

Table S1. The list of primer sequences of 6 starch synthesis gene used in interval mapping and marker regression analysis

Locus	Chr	Forward primer[a]	Reverse primer	size[b]
PUL	4	CCACCATTAAAGCATCATCAAC	AGTTGTTATATTTTAGGATGGATGG	-20
SBE4	4	CCATCACCTCAAATACATCACTC	AGACTGGAATGCCCCTTAGG	-13
Wx	6	TGTTGTTCATCAGGAAGAACATCTCCAAG	TTAATTTCCAGCCCAACACC	dCAPS(*Sty*I)
SBE1	6	TGCTACATAACACGCATACAAAGT	AGACAAAAGCGAAAGGTAATGAG	-331
ISA1	8	ATAGATGCTAATGTGATGTGGC	TGGTATAGGCACAACCGTAGA	-10
SSII-1_3	10	ATCTTTAGACGATTAGCG	AAGTCACAAGTAGAAGGG	-6

[a] primer sequences from Yan et al. (2011)

[b] the amplicon size of TNG 78 differed from TCS 17 which + and – indicate the amplicon size of TNG 78 is larger than which of TCS 17.

Table S2. The list of primer sequences of genes used in detection gene expression levels in immature grains by real-time PCR

Gene	Accession	Chr.	Forward	Reverse	Size
EFla-Q[a]	*Os03g0178000*	3	CGTTATTATCATATGCTCGCTGTGT	CAGCCGAAGGGCAATAATCA	101
SBE4[b]	*Os04g0409200*	4	GCCAATGCCAGGAAGATGA	GCGCAACATAGGATGGGTTT	128
PUL[c]	*Os04g0164900*	4	AATCTCCGATGGCTGTTCCTT	TTGAGTTCACACCCGATGCTAA	90
ISA2[b]	*Os05g0393700*	5	TAGAGGTCCTCTTGGAGG	AATCAGCTTCTGAGTCACCG	170
GBSSI[c]	*Os06g0133000*	6	GATGCGTTTCAGCCTTCTTTG	AGTATGGGTTGTTGTTGAGGTTTAG	74
SSII-3[b]	*Os06g0229800*	6	GCTTCCGGTTTGTGTGTTCA	CTTAATACTCCCTCAACTCCACCAT	54
CPE	*Os07g0604300*	7	AAACCAGGAAGCTGTGTAGA	CTGGACTAGAGGTGTCAAGC	173
AGP	*Os07g0604800*	7	TGCGATGTACTTTGGTCTTA	AGTGATCGGTAATCACCTTG	95
MADS18	*Os07g0605200*	7	ATCCCGACAACAAATAACAG	AGACGGTTGAGCTTCTGACT	66
SSII-1[c]	*Os10g0437600*	10	GATGAAGTTCCAGACGATGATGA	TGACTTAGTTGTGCCTCATAATCCT	113

[a, b, c] indicate the primer sequences adopted Jain et al. (2006), Ohdan et al. (2005), and Zeng et al. (2007), respectively.

Table S3. Descriptive statistics for eight grain quality-related physiochemical properties of rice harvested from the two crop seasons

Year and trait	Parents		RIL population	
	TNG 78	TCS 17	Mean±SD	Range
**2010-II**				
PLS	43	43	47.46±11.22	19-72
PKV (cp)	2844	2453	2369.50±396.18	1355-3595
HPV (cp)	1591	2182	1742.01±363.12	1028-2816
BDV (cp)	1253	272	654.49±453.46	5-1712
CPV (cp)	2911	4001	3313.19±683.81	2173-5115
SBV (cp)	67	1548	916.69±752.88	-566-2194
PeT (min)	5.67	6.3	6.04±0.27	5.5-6.8
PaT (℃)	86.45	87.65	88.49±2.16	74-92.9
**2011-I**				
PLS	55	ND	46.73±10.6[a]**	21-69
PKV (cp)	3066	2954	2579.10±458.14	1149-3523
HPV (cp)	1620	2556	1848.12±517.42**	605-3060
BDV (cp)	1447	398	730.98±428.08**	12-1766
CPV (cp)	2830	4486	3556.75±963.8**	1371-5488
SBV (cp)	-237	1532	985.18±813.09*	-628-2610
PeT (min)	6.03	6.17	5.95±0.3**	5.2-6.7
PaT (℃)	79.6	86.43	86.57±2.65**	75.6-91.3

[a] paired difference between the two crop seasons of 8 traits were detected by t-test which * and ** are indicated at significant levels of 0.05 and 0.01 with 2-tails, respectively.

Fig. S1.

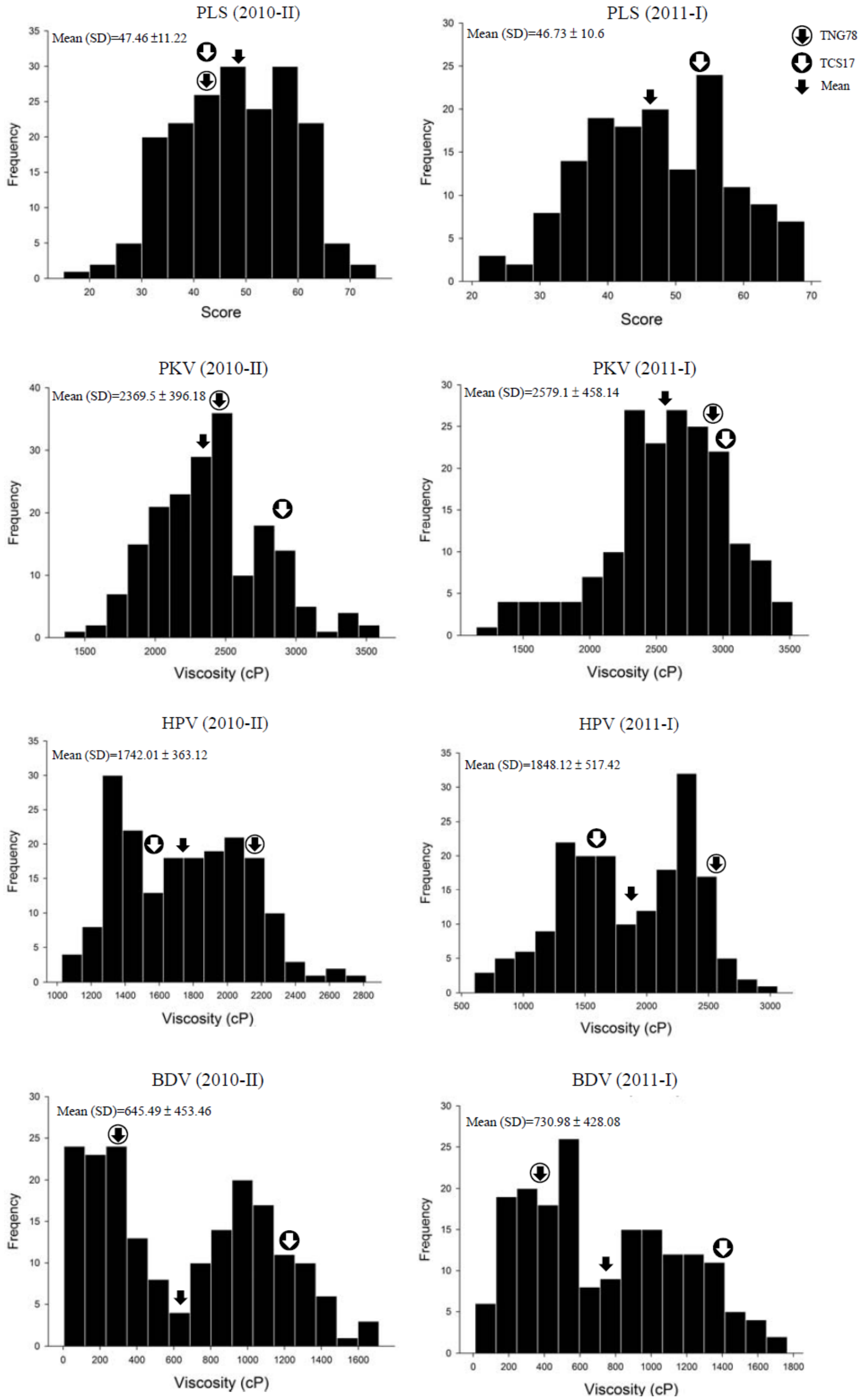

PLS (2010-II)
Mean (SD)=47.46 ±11.22
Frequency
Score
PLS (2011-I)
Mean (SD)=46.73 ± 10.6
TNG78
TCS17
Mean
Frequency
Score
PKV (2010-II)
Mean (SD)=2369.5 ± 396.18
Frequency
Viscosity (cP)
PKV (2011-I)
Mean (SD)=2579.1 ± 458.14
Freuqency
Viscosity (cP)
HPV (2010-II)
Mean (SD)=1742.01 ± 363.12
Frequency
Viscosity (cP)
HPV (2011-I)
Mean (SD)=1848.12 ± 517.42
Frequency
Viscosity (cP)
BDV (2010-II)
Mean (SD)=645.49 ± 453.46
Freqency
Viscosity (cP)
BDV (2011-I)
Mean (SD)=730.98 ± 428.08
Frequency
Viscosity (cP)

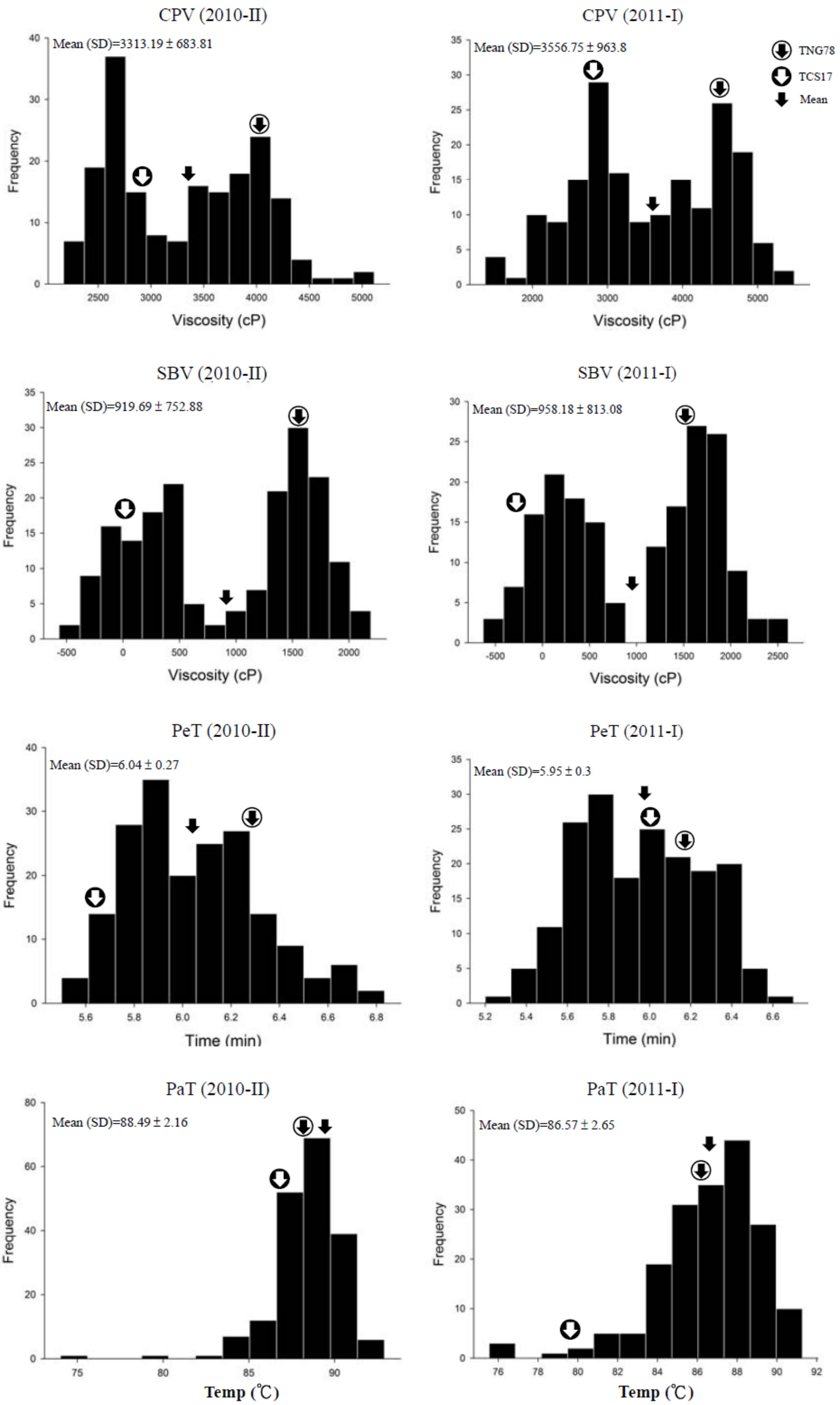

CPV (2010-II)
Mean (SD)=3313.19 ± 683.81
Frequency
Viscosity (cP)
CPV (2011-I)
Mean (SD)=3556.75 ± 963.8
TNG78
TCS17
Mean
Frequency
Viscosity (cP)
SBV (2010-II)
Mean (SD)=919.69 ± 752.88
Frequency
Viscosity (cP)
SBV (2011-I)
Mean (SD)=958.18 ± 813.08
Frequency
Viscosity (cP)
PeT (2010-II)
Mean (SD)=6.04 ± 0.27
Frequency
Time (min)
PeT (2011-I)
Mean (SD)=5.95 ± 0.3
Frequency
Time (min)
PaT (2010-II)
Mean (SD)=88.49 ± 2.16
Frequency
Temp (℃)
PaT (2011-I)
Mean (SD)=86.57 ± 2.65
Frequency
Temp (℃)
